# The Impact of Vitamin D on Immune Function and Its Role in Hashimoto’s Thyroiditis: A Narrative Review

**DOI:** 10.3390/life14060771

**Published:** 2024-06-17

**Authors:** Michela Soda, Claudia Priante, Ciro Pesce, Giovanni De Maio, Mauro Lombardo

**Affiliations:** 1 Laboratory of Clinical Research and Advanced Diagnostics, Centro di Riferimento Oncologico Della Basilicata (IRCCS-CROB), 85028 Rionero in Vulture, Italy; 2 Independent Researcher, 85100 Potenza, Italy; 3 Department of Translational Medical Sciences, School of Medicine and Surgery, University of Naples Federico II, 80131 Naples, Italy; ciropi97@gmail.com; 4 Department for the Promotion of Human Science and Quality of Life, San Raffaele Open University, Via di Val Cannuta, 247, 00166 Rome, Italy

**Keywords:** vitamin D, Hashimoto’s thyroiditis, immune system, autoantibodies, deficit, supplementation

## Abstract

Vitamin D, an essential nutrient, plays a crucial role in numerous biological functions, acting as a hormone and being important for the proper functioning of the immune system. This review illustrates the interactions between adequate vitamin D levels and an appropriate immune response, highlighting the implications for Hashimoto’s thyroiditis (HT), a chronic inflammation of the thyroid characterized by the production of autoantibodies. A comprehensive review of the existing literature shows that vitamin D inhibits the secretion of pro-inflammatory cytokines, leading to an improvement in the clinical picture in HT by switching from a pro-inflammatory to immune balance. Vitamin D supplementation has been shown to reduce elevated serum levels of thyroid peroxidase antibodies, a key marker of HT. Although the results are conflicting, the evidence suggests that an adequate vitamin D intake supports the immune function and counteracts autoimmune conditions such as HT by improving their symptoms. There is evidence of vitamin D’s key role in supporting the immune system function and managing autoimmunity, such as in HT. An adequate vitamin D intake is crucial for improving the clinical picture and the symptoms of HT.

## 1. Introduction

Hashimoto’s thyroiditis (HT) is a chronic autoimmune disease characterized by the immune system’s attack on the thyroid gland, resulting in chronic inflammation and the production of autoantibodies against specific thyroid proteins, such as thyroglobulin and thyroid peroxidase. HT is the most common cause of hypothyroidism and mainly affects women. The symptoms of HT are diverse and can include fatigue, weight gain, depression, and other signs of thyroid dysfunction [1,2,3]. The condition was first described by Dr Hakaru Hashimoto in 1912 and remains one of the most common autoimmune diseases worldwide [2].

Vitamin D, an essential nutrient, plays a key role in maintaining bone health and supporting the immune system [4,5]. It functions as a hormone, influencing various biological processes. Vitamin D regulates the immune response by modulating the activity of immune cells, including T cells, B cells, and dendritic cells. Adequate vitamin D levels are crucial for maintaining the immune balance, reducing the risk of infection, and preventing overactive immune responses that can lead to autoimmune diseases [6,7,8]. The immunomodulatory effects of vitamin D are particularly significant in the context of autoimmune diseases such as HT [9,10]. The various roles of vitamin D in different body systems and its impact on the immune system are summarized in Table 1.

This review aims to elucidate the interactions between adequate vitamin D levels and a proper immune response, with a focus on the implications for HT. The literature on this topic spans a wide range of studies exploring the role of vitamin D in the immune function and its potential impact on HT. The main objective is to synthesize current knowledge and highlight key findings that demonstrate the importance of vitamin D in the management and potential mitigation of the effects of HT. This review seeks to provide a comprehensive understanding of the existing research landscape and identify areas requiring further investigation.

Understanding the interaction between vitamin D and HT is crucial for developing effective management strategies for this autoimmune disease. Vitamin D’s role in modulating the immune response and its potential to reduce the severity of autoimmune reactions offer a promising avenue for therapeutic interventions. By clarifying how vitamin D affects the pathophysiology of HT, healthcare professionals can better tailor treatments to improve patient outcomes. This review highlights the importance of an adequate vitamin D intake in the management of HT and underlines the need for further research to fully explore its therapeutic potential.

## 2. Materials and Methods

This narrative review was conducted through a search of the scientific literature in the main electronic databases and academic search engines: PubMed, Google Scholar, and Web of Science. The search focused on the role of vitamin D in the immune system, specifically its role in the autoimmune pathology of HT.

The literature search covered publications from January 2010 to December 2023. Only articles published in English were included. The search strategy included various keywords such as “Hashimoto’s disease”, “chronic lymphocytic thyroiditis”, “Hashimoto’s disease and vitamin D”, “vitamin D in Hashimoto’s”, “thyroid peroxidase antibodies (TPOAb)”, and “thyroglobulin antibodies (TGAb)”.

After the database search, a manual review of the identified articles was conducted to improve our understanding of the clinical context and ensure the inclusion of relevant studies. Studies that emphasized the importance of vitamin D in improving the HT pathology and reducing autoantibody production were considered suitable for this review. The final selection of studies was based on the analysis of data results and graphs, which allowed us to conduct a review focusing on the potential of vitamin D in the prevention of autoimmune diseases, particularly HT, and its genetic implications. This review did not follow the PRISMA guidelines as it was a narrative review, which focused on the synthesis and interpretation of the literature rather than applying a systematic and reproducible methodology.

Data extraction was performed manually by reviewing the full texts of the selected articles. Relevant data were extracted and summarized, including study design, population characteristics, intervention details, and key findings. The synthesis process involved comparing the results between studies to identify common patterns and discrepancies. A meta-analysis was not performed due to the narrative nature of this review and the heterogeneity of the studies included.

During the manual review process, several exclusion criteria were used to ensure the inclusion of the most relevant studies. Studies whose results were not directly relevant to the role of vitamin D in the autoimmune pathology of HT were excluded. Duplicate studies were removed to avoid redundancy. In addition, studies that did not provide sufficient data on the link between vitamin D and HT were excluded. Studies that did not meet quality criteria, such as a lack of a clear methodology or unverifiable results, were excluded. Only studies published in English were included and case reports and narrative reviews were excluded, focusing mainly on original research studies.

## 3. Results

**Figure 1:**
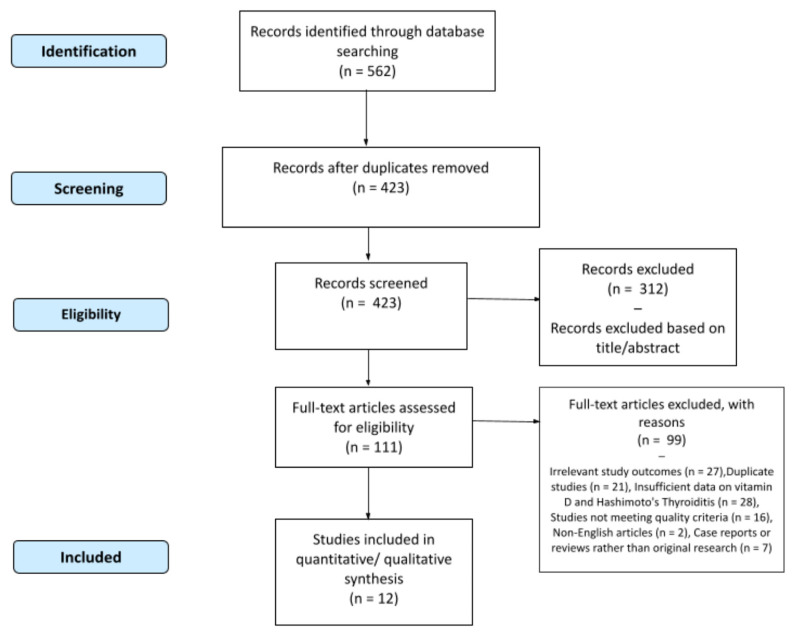
Flowchart depicting the selection process of studies included in this review.

The selection process for the studies included in this review is illustrated in the flowchart (Figure 1). A total of 562 articles were identified through the database search, and 423 articles were screened after removing duplicates. Then, 312 articles were excluded based on title and abstract screening. The remaining 111 articles were assessed for eligibility through a full-text review. After applying the inclusion and exclusion criteria, 12 articles were deemed eligible and included in the final evaluation for this review.

The bibliographic research indicates that an adequate vitamin D intake is essential not only for the optimal physiological functioning of the immune system but also for the proper functioning of various other bodily systems. Vitamin D supplementation could prevent or improve autoimmune conditions, such as HT. Indeed, many studies have highlighted a direct correlation between vitamin D deficiency and thyroid autoimmunity [24] across all age groups [25].

It seems that there are four potential mechanisms by which vitamin D contributes to the inhibition of the immune process: prevention of DC-dependent T cells’ activation and anti-inflammatory state recovery. In particular, the 1,25(OH)2D3-VDR complex can inhibit the expression of DC-secreted pro-inflammatory cytokines, thus leading to an improvement in the HT clinical picture through a switch from the Th1 to the Th2 profile as well as a decrease in the Th17-dependent pathological responses through the release of cytokines, thereby restoring the Th17/Treg ratio [25,26,27]. Nevertheless, the association between vitamin D and HT remains controversial. Many studies have analyzed this link in several populations; however, the results are still contradictory. For instance, the Croatian Biobank observations of HT patients, as well as other comparative studies, have not highlighted any direct associations between vitamin D levels and HT prevalence [28,29]. On the contrary, several large-scale studies confirm the presence of a link between low vitamin D levels and HT. Observational studies by Taheriniya et al. [30] have indeed detected lower vitamin D levels in HT patients with respect to non-HT patients, especially in female individuals [31]. Comparable results have been obtained by Štefanić and Tokić [32,33]. In addition to the association between vitamin D levels and HT prevalence, many researchers aimed to demonstrate a link between vitamin D and antithyroid antibodies. Indeed, there is a correlation between reduced levels of 25(OH)D and the presence of anti-TPO Abs [26]. A study involving 168 elderly subjects with a mean age of 82 demonstrated a significantly increased prevalence of HT and anti-TPO Abs in individuals affected by vitamin deficiency (<50 nmol/L) [25]. However, elevated serum levels of anti-TPO antibodies significantly decreased (by 20%) after a few months of vitamin D supplementation (1200–4000 IU/day) [24]. Similar results were obtained by Bozkurt et al., who demonstrated that the severeness of vitamin D deficiency was correlated with the HT duration and thyroid volume [34]. Another interesting study is the one conducted by Tokic et al., highlighting that in HT patients, T cells are characterized by decreased genetic expression of CTLA4, CD28, and CD45RAB compared to non-HT patients. These molecules appear to play a role in thyroid autoimmunity [33].

Finally, a recent meta-analysis involving eight studies (five European, two Asian, and one African) strongly correlated BsmI or TaqI VDR polymorphisms with the risk of HT, whereas ApaI and FokI polymorphisms showed no significant association. Wang et al. conducted another meta-analysis of eleven studies (five Caucasian and six Asian), which showed that the FokI VDR polymorphism was associated with the risk of developing HT only in the Asian population and not in the Caucasian population. Furthermore, the TaqI, ApaI, and BsmI polymorphisms were not positively associated in the general population, even when stratified by ethnicity [35].

Although several studies indicate a significant association between vitamin D levels and HT, there are also several studies with contradictory results. For example, observations from the Croatian Biobank and other comparative studies have shown no direct association between vitamin D levels and the prevalence of HT [28,29]. In contrast, several large-scale studies confirm a link between low vitamin D levels and HT, especially in women [30]. These discrepancies could be attributed to several factors. Population differences, such as variations in genetic background, lifestyle, and environmental factors, may influence the results. For example, the presence of VDR polymorphisms may influence the individual response to vitamin D, leading to different results in different studies. Differences in study design, sample size, and methods of measuring vitamin D levels may also contribute to inconsistent results. Some studies use serum 25(OH)D levels as a marker, while others may use different biomarkers, leading to variations in results. Confounding factors, such as age, gender, body mass index, and exposure to sunlight, may affect vitamin D levels and thyroid function. Studies that do not adequately control for these confounding factors may report different associations. Furthermore, the impact of vitamin D on HT may depend on the duration and dosage of vitamin D supplementation. Short-term or inadequately dosed studies may not fully capture the potential effect of vitamin D on thyroid health. Finally, publication bias may also play a role, as studies with significant results are more likely to be published, leading to a potential under-representation of studies with non-significant results. Considering these factors, it is clear that further research is needed to fully understand the relationship between vitamin D and HT. Future studies should aim for greater uniformity in study design, larger sample sizes, and better control of confounding variables to provide more definitive answers.

## 4. Discussion

### 4.1. Association between Vitamin D Levels and Hashimoto’s Thyroiditis

This review shows that the association between vitamin D levels and HT is supported by several studies, but also presents contradictory results. For instance, studies have shown higher levels of IL-17 and IL-23 in the group of euthyroid patients with HT, indicating immune dysregulation [26]. Furthermore, significant differences in vitamin D levels between HT patients and controls suggest that lower vitamin D levels may exacerbate the severity of HT [36,37]. Genetic factors also play a crucial role. For example, the presence of the FokI polymorphism in HT patients indicates a genetic predisposition that influences vitamin D metabolism and its impact on thyroid health [38]. Furthermore, a general vitamin D deficiency has been observed in HT patients compared to healthy controls, highlighting the need for adequate vitamin D levels in the management of HT [27,28,34,36,39]. Significant correlations between vitamin D levels and the presence of antithyroid antibodies further support the role of vitamin D in modulating immune responses in HT patients [40]. However, the heterogeneity of study designs and populations underlines the need for more standardized research to clarify these associations.

### 4.2. Genetic Factors and Population Differences

Several meta-analyses have examined the relationship between vitamin D-related genetic polymorphisms and the risk of HT. These studies provide valuable insights into the genetic basis for HT. For instance, a meta-analysis of eight studies showed a strong correlation between VDR BsmI and TaqI polymorphisms and an increased risk of HT, whereas ApaI and FokI polymorphisms showed no significant association (Table 2) [35,38]. This suggests that specific VDR polymorphisms may contribute to HT susceptibility through their influence on vitamin D metabolism and immune regulation [29]. Another meta-analysis conducted by Wang et al. on 11 studies found that the FokI VDR polymorphism was associated with HT risk in the Asian population but not in the Caucasian population [35]. This indicates potential ethnic differences in the genetic predisposition to HT, highlighting the need for population-specific studies to better understand these genetic influences. These results underline the importance of genetic factors in the pathogenesis of HT. The association between certain VDR polymorphisms and the HT risk suggests that genetic variations may influence the efficacy of vitamin D in modulating immune responses and maintaining thyroid health [29]. Understanding these genetic differences is crucial for developing personalized approaches to the prevention and treatment of HT, which could involve targeted vitamin D supplementation based on the individual’s genetic profile.

### 4.3. Mechanisms of Vitamin D in Autoimmune Regulation

Numerous studies have shown that there is a direct correlation between vitamin D deficiency and thyroid autoimmunity in all age groups. There are two mechanisms by which this vitamin contributes to the inhibition of the immune process: prevention of DC-dependent T-cell activation and restoration of the anti-inflammatory state; down-regulation of HLA class II gene expression within the thyroid, which also affects the B-cell response [41,42]. The 1,25(OH)2D3-VDR complex succeeds in inhibiting the expression of pro-inflammatory cytokines by DCs, thus favoring an improvement in the clinical picture of HT through a shift from a Th1 to a Th2 profile, as well as a decrease in pathological responses by Th17 and restoration of the Th17/Treg ratio [24,26]. Furthermore, there are differences between the HT and non-HT population, especially with regard to follicular cells expressing MHC class II, which are crucial for the production of autoantigens and T-cell activation, a process in which IFN-γ and IL-12 participate [43]. However, it is known that 1,25(OH)2D can reduce MHC II expression [39,42,44].

Although the association between low vitamin D levels and HT remains controversial, many studies confirm it, especially in women [31,39,44]. Similar results were obtained by Štefanić, Tokić, Wang, and colleagues [32,33,35]. Furthermore, a correlation between low vitamin D levels and the presence of anti-TPO Abs has been demonstrated. Elevated serum levels of anti-TPO antibodies strongly decreased after several months of vitamin D supplementation [24,40]. HT duration and thyroid volume were both found to be correlated with the degree of deficiency [34]. Tokić et al. point out that, in HT patients, T cells are characterized by lower gene expression of CTLA4, CD28, and CD45RAB than in non-HT patients, suggesting that these molecules could potentially be involved in autoimmunity [33].

## 5. Conclusions

This review highlights the importance of micronutrients in the immune system, particularly vitamin D. A correct and balanced diet provides the human body with molecules essential for energy and defense mechanisms, such as vitamin D. Vitamin D interacts directly with the epigenome by regulating transcription factors and remodeling chromatin, promoting important responses to adverse events. Consequently, vitamin D has been called an ‘ally of the immune system’. An adequate intake of vitamin D promotes responses to cardiovascular, bone, brain, inflammatory, and autoimmune diseases, including HT.

Recent scientific research supports the hypothesis that vitamin D exerts immunomodulatory and anti-inflammatory effects on HT by preventing the activation of T cells dependent on dendritic cells and inhibiting the release of pro-inflammatory cytokines. Vitamin D improves the clinical picture of patients with HT by reducing the levels of autoantibodies directed against the thyroid gland. Although the results of the extant studies vary, perhaps due to the expression of different polymorphisms, the importance of sufficient and proper supplementation is evident, showing great promise and encouraging further research in this direction.

Although vitamin D supplementation induces changes in the amount of anti-TPO antibodies in HT patients, no significant correlation has been found between serum vitamin D and thyroid antibodies, TSH, fT3, and fT4. Numerous preclinical and observational studies highlight the importance of adequate vitamin D supplementation, demonstrating its potential benefits and encouraging further research in this field. In sum, it seems that maintaining adequate vitamin D levels is crucial for the management of autoimmune diseases such as HT, and further studies are needed to fully understand its therapeutic potential.

## Figures and Tables

**Table 1:** The role of vitamin D in various body systems and the immune system.

[Ref]	Consequences of the Deficit	Vitamin D Role	Apparatus or System
[4,5]	Bone deformation, stunted growth, osteomalacia, delayed mineralization	Bone health and metabolism, mineralization	Skeletal
[11]	Dry and brittle skin	Linked to skin health via keratinocyte function and immune modulation	Integumentary
[12]	Hypertension, atherosclerosis	Prevention of chronic cardiovascular diseases	Cardiovascular
[13,14]	Depression, stress, bad mood	Mood regulation	Depression and anxiety
[7,8]	Increased risk of infections	Activation of pathogen recognizing TLR receptors; Stimulation of antimicrobial activity by macrophages/monocytes with VDR-RXR signaling; Increased production of IL-1	Innate/Adaptive immune system: Bacterial infection
[15,16]		Increased Tregs and Th2 cells	
[17,18,19,20,21,22]		Pro-inflammatory M1→M2 shift	
[9,10]		Interaction with APC and differentiation of Tregs	
[23]	Limited risk of autoimmune diseases	Action on HLA	

This table summarizes the functions of Vitamin D in different body systems and the immune system, outlining the consequences of Vitamin D deficiency. Abbreviations: Ca^2+^: calcium ion, IL: interleukin, MHC: major histocompatibility complex, RXR: retinoid X receptor, Th: T-helper cells, TLR: Toll-like receptor, Tregs: regulatory T cells, VDR: vitamin D receptor.

**Table 2:** Summary of clinical studies on vitamin D and Hashimoto’s thyroiditis.

Results	Study and Methodology	Control	HT Patients	Study Focus	Year	Author
Significant differences correlated with the presence of antithyroid Abs (*p* = 0.01) and abnormal thyroid function tests (*p* = 0.059)	LIAISON chemiluminescence immunoassay	42	50	Presence of antithyroid antibodies	2011	Kivity et al. [40]
Serum vitamin D levels directly correlated with thyroid volume (*p* < 0.001) and inversely correlated with anti-TPO (*p* < 0.001) and anti-TG levels (*p* < 0.001)	Thyroid ultrasound, evaluation of vitamin D serum, anti-TPO, and anti-TG	180	180	Vitamin D and HT pathogenesis	2013	Bozkurt et al. [34]
Significant negative correlation between vitamin D levels and anti-TPO (*p* < 0.0001) and anti-TG (*p* < 0.001)	HT patients received vitamin D3 orally for 4 months (1200–4000 IU)	NA	218	Vitamin D and HT pathogenesis	2015	Mazokopakis et al. [24]
Higher levels in euthyroid HT group (*p* = 0.041 for IL-17 and *p* < 0.001 for IL-23)	Study of IL-17 and IL-23 levels	26	46	Interleukin levels	2016	Konca et al. [27]
Higher incidence of thyroid diseases in subjects with vitamin D deficiency (*p* = 0.002). No correlation between vitamin D levels and TG-Ab (*p* = 0.25)	NA	168	NA	Thyroid diseases and vitamin D deficiency	2016	Muscogiuri et al. [26]
Higher expression levels of VDR CTLA4 CD28 CD45RAB mRNA in T-lymphocytes of healthy controls compared to HT (no *p* values)	NA	13	45	Altered CTLA-4 expression	2017	Tokić et al. [33]
No significant correlation between BsmI polymorphism (*p* = 0.727), ApaI polymorphism (*p* = 0.869), and HT risk	VDR genotyped by PCR and MALDI-TOF-MS	1303	1338	VDR gene polymorphisms and HT risk	2017	Wang et al. [35]
General deficiency of vitamin D in HT patients (*p* = 1.5 × 10^−5^) compared to healthy controls (*p* = 5.7 × 10^−6^)	NA	2263	2695	General vitamin D levels	2020	Štefanić et al. [32]
Significant differences in vitamin D levels between subgroups (*p* = 0.023)	Vitamin D measured from stored serum samples	176	461	Severity of HT related to vitamin D levels	2021	Cvek et al. [28]
Higher presence of FokI in HT patients (*p* = 0.02); no significant difference in serum vitamin D levels between HT and control (*p* = 0.223)	Serum vitamin D analyzed using HPLC-UV method	48	272	Vitamin D deficiency in HT and implications	2021	Hanna et al. [29]
Elevated TSH levels in the majority; lower vitamin D levels in primary hypothyroidism; negative association between TSH and vitamin D in women <45 years (*p* = 0.036). Significant negative association between heterogeneous thyroid parenchyma and vitamin D in women (*p* = 0.048)	NA	NA	1295	HT in women	2021	Turashvili et al. [31]
No significant differences in patients and control groups. No significant correlation between vitamin D and thyroid autoantibodies	Vitamin D, thyroid autoantibodies (a-TPO, a-TG), free T4 and TSH levels	41	57	HT in in premenopausal women	2023	Filipova et al. [36]

The table shows key findings from various studies on the relationship between vitamin D and Hashimoto’s thyroiditis. Abbreviations: anti-TPO: antithyroid peroxidase antibody, HT: Hashimoto’s thyroiditis, HPLC-UV: high-performance liquid chromatography with ultraviolet detection, IL: interleukin, MALDI-TOF-MS: matrix-assisted laser desorption/ionization time-of-flight mass spectrometry, NA: data not available, PCR: polymerase chain reaction, TG-Ab: thyroglobulin antibody, TSH: thyroid-stimulating hormone, VDR: vitamin D receptor.

## Data Availability

Not applicable.
